# Predictive value of arterial blood lactate/serum albumin ratio for myocardial injury in elderly patients with severe community-acquired pneumonia

**DOI:** 10.1097/MD.0000000000028739

**Published:** 2022-01-28

**Authors:** Litao Zhang, Yanpeng Li, Chang Lv, Hui Guo, Tieling Xu, Zhichao Ma, Jianguo Li

**Affiliations:** Department of Emergency, Hebei General Hospital, Shijiazhuang Hebei, China.

**Keywords:** lactate/albumin, myocardial injury, severe community-acquired pneumonia

## Abstract

To investigate the predictive value of arterial blood lactate (Lac)/serum albumin (Alb) ratio (Lac/Alb) on myocardial injury in elderly patients with severe community-acquired pneumonia (SCAP).

Seventy-two elderly SCAP patients hospitalized in the intensive care unit (ICU) of the emergency department of Hebei General Hospital from March 2020 to March 2021 were included, and the general data and arterial blood Lac and serum Alb levels were collected, and Lac/Alb values were calculated. The patients were divided into myocardial injury group (n = 25) and nonmyocardial injury group (n = 47) according to whether the myocardial injury occurred during their ICU stay, and the predictive value of Lac/Alb on myocardial injury in elderly patients with SCAP was assessed using receiver operating characteristic curve and area under the curve.

There were no statistically significant differences in age and gender between the 2 groups (both *P* > .05), and there were no statistical differences in oxygenation index, procalcitonin, C-reactive protein, lymphocyte count, and Alb levels between the 2 groups (all *P* > .05). Neutrophil count, neutrophil\lymphocyte ratio, serum creatinine, Lac, and Lac/Alb levels were significantly higher in patients in the myocardial injury group than in the nonmyocardial injury group [13.90 (11.07,19.67) × 10^9^/L vs 10.79 (8.16,14.23) × 10^9^/L, 26.48 (20.07,31.88) vs 17.79 (9.85,27.23), 135.71 (81.50,284.75) μmol/L vs 76.30 (60.30,140.30) μmol/L, 3.0 (2.2,4.5) mmol/L vs 2.1 (1.6,3.1) mmol/L, 1.34 (0.88,2.16) vs 0.78 (0.60,1.12), all *P* < .05]. Patients in the myocardial injury group had a significantly higher mortality rate in the ICU than in the nonmyocardial injury group (72.0% vs 36.2%, *P* < .01). Neutrophils, neutrophil/lymphocyte ratio, serum creatinine, Lac, and Lac/Alb showed a weak positive correlation with myocardial injury in patients (all *P* < .05). The area under the curve of Lac/Alb for predicting myocardial injury in elderly patients with SCAP was 0.737 (95% confidence interval 0.620–0.834), and the sensitivity and specificity of the prediction with 1.21 as the cutoff value were 60.00% and 78.72%, respectively.

Lac/Alb has an excellent predictive value for myocardial injury in elderly SCAP patients.

## 1 Introduction

Community-acquired pneumonia (CAP) which is predominately caused by Streptococcus pneumoniae is one of the most widespread and fatal infectious pathologies. The annual incidence of CAP diagnosed varies between different communities. It accounts for approximately 3 million deaths per year worldwide.^[1]^ CAP, especially severe community-acquired pneumonia (SCAP) is a leading infectious cause of hospitalization and death among adults admitted to the intensive care unit (ICU).^[2]^ A sizeable population-based surveillance study on hospitalized CAP patients found that 21% of patients required ICU admission, with 26% of them needing mechanical ventilation.^[3]^ SCAP is a progressive disease, from local to systemic inflammation of the lung, with a vicious onset and rapid progression, often leading to acute respiratory failure, with some patients combining septic shock, multiple organ dysfunction syndrome, and other complications. SCAP hospital mortality is high, ranging from 25% to more than 50%.^[4,5]^ The mortality of CAP increases with the patient's age, and the mortality of elderly patients with SCAP can reach more than 50%.^[6]^ SCAP patients are often complicated by myocardial injury^[7]^ and even septic cardiomyopathy (SCM), which aggravates circulatory dysfunction, with a mortality rate of up to 70%.^[8]^ Therefore, predicting myocardial injury in SCAP patients is clinically significant for the early detection and timely management of the disease. Lactate (Lac) is an essential indicator of inadequate tissue perfusion and cellular hypoxia, and patients with sepsis have varying degrees of tissue hypoperfusion and impaired oxygenation, which leads to increased anaerobic metabolism and the production of large amounts of Lac. Current studies have confirmed that Lac is a reliable predictive biomarker for developing multiple organ dysfunction and adverse outcomes in patients with sepsis.^[9]^ Albumin (Alb) has multiple physiological functions such as antioxidant, anti-inflammatory, and vascular endothelial functional integrity maintenance. Alb levels can reliably reflect frailty, susceptibility to stressors, and unstable health status and are associated with the prognosis of severe disease.^[10]^ The single value of Lac and Alb levels in patients with sepsis is well established, and the Lac to Alb ratio (Lac/Alb) may be more valuable in infectious diseases. Therefore, in this study, we aimed to investigate the predictive value of Lac/Alb on myocardial injury in elderly patients with SCAP.

## 2 Materials and methods

### 2.1 Study subjects

This was a single-center retrospective study in which 72 elderly SCAP patients met the inclusion criteria, including 55 males and 17 females, who were admitted to the ICU of the emergency department of the Hebei General Hospital from March 2020 to March 2021. Patients were divided into myocardial injury group (n = 25) and nonmyocardial injury group (n = 47) according to whether the myocardial injury occurred during their ICU stay. Myocardial injury was defined as a troponin T (TnT) level exceeding the upper limit of the standard reference value (99th percentile as the upper limit of the reference value), and acute coronary syndrome (ACS) was excluded according to the patients’ medical history, symptoms, electrocardiogram, echocardiogram, and relevant laboratory tests. The study was performed in strict compliance with the protocol approved by the Ethics Committee of the Hebei General Hospital.

Inclusion criteria: age ≥ 65 years, diagnostic criteria for SCAP refer to the Infectious Diseases Society of America/American Thoracic Society guidelines for the diagnosis and management of CAP in adults,^[11]^ no previous history of myocardial injury, and complete data.

Exclusion criteria: immunocompromised patients such as after solid organ transplantation and hematopoietic stem cell transplantation, active malignancy, receiving cancer chemotherapy, treated with corticosteroids at ≥20 mg prednisone daily or equivalent dose for more than 14 consecutive days, etc^[12]^; combined acute and chronic heart failure, ACS, pulmonary embolism, aortic coarctation, cardiac valve disease, cardiomyopathy, pericarditis, end-stage renal disease, and acute cerebrovascular disease; history of cardiac surgery, cardiopulmonary resuscitation in the last 3 months; patients with poisoning; women in pregnancy; incomplete data; and patients in palliative care.

### 2.2 Methods

Emergency physicians gave early treatment to enrolled patients, including mechanical ventilation, fluid resuscitation, administration of vasoactive drugs, and anti-infection, referring to the Chinese guidelines for the diagnosis and treatment of CAP in adults (2016 edition)^[13]^ and the international guidelines for the management of sepsis and septic shock (2016),^[14]^ respectively. Venous and arterial blood were drawn separately to test the biochemical parameters of patients using Roche automatic biochemical analyzer and supporting reagents, and arterial blood gas was measured using an automatic blood gas analyzer. The main information collected from all subjects included gender, age, oxygenation index, procalcitonin, C-reactive protein, neutrophil count, lymphocyte count, serum creatinine (Scr), Alb levels, and Lac levels. Neutrophil\lymphocyte ratio (NLR) and Lac/Alb were calculated. After admission to the emergency ICU, the patients were treated according to the guidelines for maintaining hemodynamic stability, mechanical ventilation, sputum drainage, antimicrobial therapy, nutritional support, and treatment of underlying diseases. Serum TnT levels were measured and dynamically monitored for changes during treatment using enzyme-linked fluorescence analysis. Patients were grouped according to the definition of myocardial injury and finally counted for mortality in the ICU.

### 2.3 Statistical analysis

The data were processed and analyzed by SPSS 26.0 software (IBM). Normally distributed measurement data were expressed as mean ± standard deviation ($\bar{X}\pm \text{S}$), and independent samples *t* test was used to compare means between groups. Nonnormally distributed continuous variables were expressed as median (first, third quartiles), and the Mann–Whitney *U* test was used for the comparison of means between groups. Correlations between the 2 groups of measurement data were analyzed using Spearman correlation analysis. The counting data were expressed by frequency and rate, and the comparison between groups was performed by χ^2^ test.

MedCalc 12.7.0 software (Ostend, Belgium) was used to calculate the receiver operating characteristic curve and the area under the curve (AUC). Z test was used to compare the 2 AUCs. The Youden index was calculated, and the value at the maximum Youden index was used as the cutoff value. The sensitivity, specificity, positive likelihood ratio, and negative likelihood ratio were determined according to the cutoff value. *P* < .05 was considered to be statistically significant.

## 3 Results

### 3.1 Comparison of general clinical information between 2 groups

There were no statistically significant differences in age and gender between the 2 groups (both *P* > .05), and there were no statistical differences in oxygenation index, procalcitonin, C-reactive protein, lymphocyte count, and Alb levels between the 2 groups (all *P* > .05). Neutrophil count, NLR, Scr, Lac, and Lac/Alb levels were significantly higher in patients in the myocardial injury group than in the nonmyocardial injury group (all *P* < .05). Patients in the myocardial injury group had significantly higher mortality in the ICU than in the nonmyocardial injury group (*P* < .01) (Table 1).

**Table 1:** Results of general clinical data of patients in both groups [$\bar{X}\pm \text{S}$, M (Q_L_,Q_U_), n (%)].

	Myocardial injury group (n = 25)	Nonmyocardial injury group (n = 47)	*t/*χ^2^/Z	*P*
Age	74.08 ± 14.36	71.40 ± 12.11	0.836	.406
Males	19 (76.0)	36 (76.6)	0.003	.955
PaO_2_/FiO_2_ (mm Hg)	152.67 (94.17,198.12)	157.60 (106.33,214.29)	−0.775	.438
PCT (ng/mL)	22.38 ± 28.17	14.48 ± 24.76	1.228	.224
CRP (mg/L)	173.32 (91.56,226.69)	154.19 (97.98,260.61)	−0.408	.683
Neutrophil (×10^9^/L)	13.90 (11.07,19.67)	10.79 (8.16,14.23)	−2.525	.012
Lymphocyt (×10^9^/L)	0.57 (0.44,0.74)	0.62 (0.32,0.92)	−0.225	.822
NLR	26.48 (20.07,31.88)	17.79 (9.85,27.23)	−2.584	.010
Lac (mmol/L)	3.0 (2.2,4.5)	2.1 (1.6,3.1)	−3.235	.001
Alb (g/dL)	2.76 (2.13,3.12)	2.69 (2.47,2.92)	−0.491	.623
Lac/Alb	1.34 (0.88,2.16)	0.78 (0.60,1.12)	−3.288	.001
Scr (μmol/L)	135.71 (81.50,284.75)	76.30 (60.30,140.30)	−2.856	.004
Death	18 (72.0)	17 (36.2)	8.387	.004

CRP = C-reactive protein, Lac = lactate, Lac/Alb = lactate/albumin ratio, NLR = neutrophil/lymphocyte ratio, PaO_2_/FiO_2_ = oxygenation index, PCT = procalcitonin, Q_L_,Q_U_ = first/third quartile, Scr = serum creatinine.

### 3.2 Spearman correlation analysis of factors associated with myocardial injury in elderly patients with SCAP

Neutrophils, NLR, Scr, Lac, and Lac/Alb showed a weak positive correlation with myocardial injury in elderly patients with SCAP (Table 2).

**Table 2:** Results of Spearman analysis of factors associated with myocardial injury in elderly patients with SCAP.

	*r*_s_	*P*
PaO_2_/FiO_2_	−0.092	.442
PCT	0.187	.115
CRP	−0.048	.686
Neutrophil	0.300	.011
Lymphocyte	−0.027	.824
NLR	0.307	.009
Lac	0.384	.001
Alb	0.058	.627
Lac/Alb	0.390	.001
Scr	0.339	.004

Alb = albumin, CRP = C-reactive protein, Lac = lactate, Lac/Alb = lactate/albumin ratio, PaO_2_/FiO_2_ = oxygenation index, NLR = neutrophil/lymphocyte ratio, PCT = procalcitonin, SCAP = severe community-acquired pneumonia.

### 3.3 Optimal cutoff values and sensitivity and specificity of Lac/Alb for predicting myocardial injury in elderly patients with SCAP

**Figure 1:**
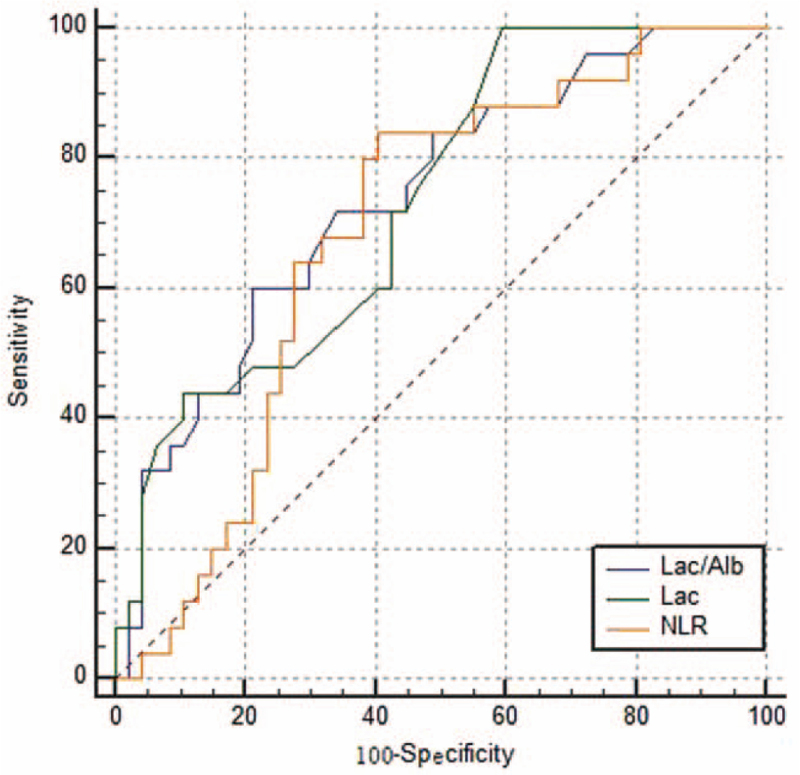
ROC curves of Lac/Alb, Lac, and NLR for predicting myocardial injury in elderly patients with SCAP. Lac/Alb = lactate/albumin ratio, Lac = lactate, NLR = neutrophil/lymphocyte ratio, ROC = receiver operating characteristic.

The AUCs of Lac/Alb, Lac, and NLR for predicting myocardial injury in patients with SCAP were 0.737 [95% confidence interval (CI) 0.620–0.834], 0.732 (95% CI 0.615–0.830) and 0.686 (95% CI 0.566–0.790). Pairwise comparisons did not show significant differences among the 3 groups (all *P* > .05) (Fig. 1, Table 3).

**Table 3:** Optimal cutoff values and sensitivity and specificity of Lac/Alb, Lac, and NLR for predicting myocardial injury in elderly patients with SCAP.

	Cutoff value	Sensitivity	Specificity	Positive likelihood ratio	Negative likelihood ratio
Lac/Alb	1.21	60.00 (95% CI: 38.7–78.9)	78.72 (95% CI: 64.3–89.3)	2.82	0.51
Lac (mmol/L)	1.9	100.00 (95% CI: 86.3–100.0)	40.43 (95% CI: 26.4–55.7)	1.68	0.00
NLR	18.6	84.00 (95% CI: 63.9–95.5)	59.57 (95% CI: 44.3–73.6)	2.08	0.27

CI = confidence interval, Lac = lactate, Lac/Alb = lactate/albumin ratio, NLR = neutrophil/lymphocyte ratio , SCAP = severe community-acquired pneumonia.

## 4 Discussion

Currently, the mechanism of SCAP is unclear and incompletely understood. The efforts of researchers are aimed at identifying the CAP severity in patients such as those who are prone to combine myocardial injury. This study showed that neutrophil count, NLR, Scr, Lac, and Lac/Alb levels were significantly higher in the myocardial injury group than in the nonmyocardial injury group (all *P* < .05). NLR, Lac, and Lac/Alb were weakly positively correlated with myocardial injury in elderly patients with SCAP. Lac/Alb was better than Lac and NLR in predicting myocardial injury in elderly patients with SCAP, and the value of the 3 AUC were 0.737, 0.732, and 0.686, respectively, but there were no significant differences among the 3 groups (all *P* > .05).

The fate of individual organs in sepsis is interdependent: failure of 1 organ often leads to dysfunction or failure of other organs.^[15]^ Patients with severe pneumonia often have combined cardiac involvement, manifesting as myocardial injury or even SCM. There is no uniform definition or diagnostic criteria for SCM. SCM can be broadly defined as a syndrome of acute cardiac insufficiency unrelated to cardiac ischemia in patients with sepsis. Its manifestations include left and/or right ventricular impairment during systole or diastole, inadequate cardiac output and oxygen delivery, or primary myocardial cellular injury.^[16]^ As there are not enough data to provide a uniform diagnosis, this study used TnT levels above the upper limit of normal reference values and excluded ACS, defined as myocardial injury, meanwhile included SCM.

The etiology of myocardial injury due to sepsis is complex, in which the dysregulated inflammatory response caused by sepsis is directly related to myocardial cell dysfunction. In this study, neutrophils and NLR were significantly higher in patients in the myocardial injury group than in the nonmyocardial injury group, suggesting to some extent an association with the inflammatory response. At present, NLR has been recognized as one of the effective indicators of systemic inflammation. More and more evidence shows that NLR is a simple and practical detection index, which can accurately diagnose bacterial pneumonia and evaluate the severity and prognosis of patients. A recent study in patients with novel coronavirus pneumonia has shown that NLR has an excellent predictive value for myocardial injury in heavy novel coronavirus pneumonia patients (AUC = 0.774, 95% CI 0.694–0.842), with an optimal cutoff value of 5.768, a sensitivity of 82.8%, and specificity of 69.5%.^[17]^ In this study, it was found that NLR could also predict the occurrence of myocardial injury in elderly patients with SCAP (AUC = 0.686, 95% CI 0.566–0.790). Due to different study populations and types of pathogens, the change degree of neutrophils and lymphocytes after pulmonary infection differed, so the predictive value was different.

Alb levels have been found to fall sharply in severe infections (eg, bacteremia) and correlate with poor prognosis.^[18]^ However, Alb levels are also affected by the nutritional status of the patient and chronic inflammation, and serum Alb levels in patients with liver dysfunction are often lower than normal due to hepatic synthesis of Alb.^[19]^ Therefore, there are limitations in predicting patient prognosis based on Alb levels alone. Lac elevation may be due to decreased clearance, increased production, or both. Low perfusion status is a common cause of increased Lac, but it is also affected by various other factors, such as certain tumors, intestinal ischemia, thiamine deficiency, mitochondrial damage, drug effects (such as metformin).^[20]^ Thus the pathophysiological mechanisms leading to Lac elevation are complex. Combining Lac and Alb or Lac/Alb may be a better clinical predictor for patients with severe infections.

The clinical value of Lac/Alb in predicting prognosis in critically ill patients has been confirmed by some studies. Gharipour et al^[21]^ found that Lac/Alb was associated with ICU and in-hospital mortality and performed comparably to Lac in predicting 28-day mortality in patients with severe infections. However, some studies have found that the Lac/Alb is better than Lac and Alb alone (AUC = 0.67, 0.61, and 0.34, respectively) in predicting hospital mortality in adult sepsis patients.^[22]^ A new observational study found Lac/Alb was an independent risk factor for death in patients with sepsis, and Lac/Alb × age score was more accurate and convenient for providing a general assessment of prognosis.^[23]^ This study found that the Lac/Alb could also predict myocardial injury in elderly patients with SCAP (AUC = 0.737, 95% CI 0.620–0.834), which was better than Lac and NLR, but there were no statistical differences between the 3. The diagnostic sensitivity of Lac was 100%, but the specificity was only 40.43%, which limited its clinical application.

The study found that risk factors for SCM included younger age (odds ratio [OR] = 0.97, 95% CI 0.95–0.99), higher Lac levels at admission (OR = 1.18, 95% CI 1.05–1.32), and a history of heart failure (OR = 3.77, 95% CI 1.37–10.40). Also there were no significant differences in in-hospital mortality and 30-day mortality between SCM and non-SCM patients (24.1% vs 12.7%, *P* = .15; 20.7% vs 12.1%, *P* = .23).^[24]^ There were no differences in gender and age between the 2 groups in this study, and Lac levels were significantly higher in patients in the myocardial injury group than in the nonmyocardial injury group (*P* < .01), which was the same as the above results. However, this study showed higher mortality in the myocardial injury group (*P* < .01). The prognosis of patients with SCM is unclear, possibly because of the different diagnostic criteria used. In a study analyzing left ventricular ejection fraction and 30-day mortality in patients with severe sepsis or septic shock, left ventricular ejection fraction was not found to be a sensitive or specific predictor of mortality.^[25]^

In conclusion, Lac/Alb has a clinical predictive value for myocardial injury in elderly SCAP patients, superior to Lac and NLR, and can be used as a new method for clinical assessment. Because SCAP is a rapidly developing disease, dynamic observation of the changes in the biomarker levels is of particular interest. It can be a useful auxiliary tool for the prompt selection of individual therapies for CAP.

This study has a few limitations. First, the sample size was small. Therefore, the clinical value of Lac/Alb needs to be verified by clinical studies with a larger sample size. Second, the Lac/Alb was measured only at a one-time point. Further study should investigate the changes over time and the serial cutoff values according to the outcome. Third, no healthy control and CAP groups were included in this study, and there were no comparisons with SCAP patients.

## Acknowledgments

We would like to thank Litao Zhang, Yanpeng Li, Chang Lv, Hui Guo, Tieling Xu, Zhichao Ma, and Jianguo Li for their assistance and valuable discussion.

## Author contributions

**Data curation:** Tieling Xu, Zhichao Ma.

**Investigation:** Yanpeng Li.

**Methodology:** Chang Lv.

**Project administration:** Litao Zhang.

**Writing – original draft:** Hui Guo.

**Writing – review & editing:** Jianguo Li.
